# Low-cost carbon-silicon nanocomposite anodes for lithium ion batteries

**DOI:** 10.1186/1556-276X-9-360

**Published:** 2014-07-18

**Authors:** Nacer Badi, Abhinay Reddy Erra, Francisco C Robles Hernandez, Anderson O Okonkwo, Mkhitar Hobosyan, Karen S Martirosyan

**Affiliations:** 1 Center for Advanced Materials, University of Houston, Houston, TX 77204-5004, USA; 2 Department of Physics, College of Science, University of Tabuk, P.O. Box 741, Tabuk 71491, Kingdom of Saudi Arabia; 3 College of Engineering Technology, University of Houston, Houston, TX 77204-4020, USA; 4 Department of Physics and Astronomy, University of Texas at Brownsville, Brownsville, TX 78520, USA

**Keywords:** Nanostructured carbon, Carbon-silicon nanocomposite, Anode materials, Lithium ion batteries, Electrochemical energy storage, Ball milling technique

## Abstract

The specific energy of the existing lithium ion battery cells is limited because intercalation electrodes made of activated carbon (AC) materials have limited lithium ion storage capacities. Carbon nanotubes, graphene, and carbon nanofibers are the most sought alternatives to replace AC materials but their synthesis cost makes them highly prohibitive. Silicon has recently emerged as a strong candidate to replace existing graphite anodes due to its inherently large specific capacity and low working potential. However, pure silicon electrodes have shown poor mechanical integrity due to the dramatic expansion of the material during battery operation. This results in high irreversible capacity and short cycle life. We report on the synthesis and use of carbon and hybrid carbon-silicon nanostructures made by a simplified thermo-mechanical milling process to produce low-cost high-energy lithium ion battery anodes. Our work is based on an abundant, cost-effective, and easy-to-launch source of carbon soot having amorphous nature in combination with scrap silicon with crystalline nature. The carbon soot is transformed *in situ* into graphene and graphitic carbon during mechanical milling leading to superior elastic properties. Micro-Raman mapping shows a well-dispersed microstructure for both carbon and silicon. The fabricated composites are used for battery anodes, and the results are compared with commercial anodes from MTI Corporation. The anodes are integrated in batteries and tested; the results are compared to those seen in commercial batteries. For quick laboratory assessment, all electrochemical cells were fabricated under available environment conditions and they were tested at room temperature. Initial electrochemical analysis results on specific capacity, efficiency, and cyclability in comparison to currently available AC counterpart are promising to advance cost-effective commercial lithium ion battery technology. The electrochemical performance observed for carbon soot material is very interesting given the fact that its production cost is away cheaper than activated carbon. The cost of activated carbon is about $15/kg whereas the cost to manufacture carbon soot as a by-product from large-scale milling of abundant graphite is about $1/kg. Additionally, here, we propose a method that is environmentally friendly with strong potential for industrialization.

## Background

The current electrochemical-based energy storage technology uses primarily activated carbon (AC) electrodes for their intended applications, which are indeed cost effective and scalable, but seriously lacks performance for higher specific capacity. Carbon nanostructures (CNSs) composed of CNT, graphene, and carbon nanofibers come with outstanding properties and are the most sought alternatives to replace AC materials but their synthesis cost makes them cost-prohibitive. Most importantly, using graphene or graphene oxide requires complex, tedious, and in some cases toxic processes [1,2]. In addition, some synthesis processes represent serious health concern [3-6]. Silicon has recently emerged as a strong candidate to replace existing graphite anodes due to its inherently large theoretical gravimetric specific capacity of ~4,200 mAh/g and low working potential at around 0.5 V. This is based on the formation of the Li_4.4_Si alloy, which is ten times higher than that of conventional carbon anodes (~372 mAh/g corresponding to the formation of LiC_6_) [7-12].

The use of silicon anodes in Li^+^ battery systems has been limited by rapid capacity degradation after only a few charge-discharge cycles. The drastic volume change (larger than 300%) upon lithium alloying/de-alloying reactions with Si commonly causes rapid decrease in reversible capacity and a continuous formation of the so-called solid-electrolyte interphase (SEI) as a result of silicon pulverization. Although various advances employing porous silicon, silicon nanoparticles, and silicon-coated carbon nanofibers have been investigated, they have shown limited improvements in cycling stability and capacity [13-20]. In these materials, a highly conductive porous carbon framework provides a mechanical support for Si nanoparticles and an electrical conducting pathway during the intercalation process of lithium ions. The poor capacity retention and low power density remain two unsolved challenges in silicon-based anode technologies. A recent research progress by Hui Wu et al. using double-walled silicon nanotube (DWSiNT) anodes for LIBs reported 6,000 electrochemical cycles, while retaining more than 85% of the initial capacity [21]. Although elaborated DWSiNT anode materials offer high specific capacity and excellent capacity retention that lasts far more what is needed by electric vehicles, the practical application is hampered because of the synthesis method used is costly and time consuming for the industry.

In this manuscript, we report on the synthesis and use of carbon and hybrid carbon-silicon nanostructures made by a simplified thermomechanical milling process to produce low-cost high-energy lithium ion battery anodes. The carbon-silicon nanocomposites provide excellent mechanical, electrochemical, and electrical properties of carbon with the superior lithium intercalation ability of silicon. The manufacturing of carbon-silicon composites for anodes by mechanical milling has been successfully explored [22-27]. Regardless of the efforts, the anodes are fading [23,14]. One of the main reasons is directly related to the mechanical integrity of the composite materials [28]. Most researchers ignore the importance of mechanical properties in the anodes that may be the single most important property to prevent the well-known fading in the specific capacity of carbon-silicon composites. In this work, we used a source of carbon that can be processed mechanically and that can be used to coat the silicon particles increasing their mechanical electrical properties.

## Methods

### Material processing

The fullerene soot is produced by the Kratschmer method and is the by-product obtained after the purification of fullerene [29]. The soot used in the present work has less than 1 wt% fullerenes (C_60_ and C_70_). The presence of fullerenes is observed by characterization methods such as X-ray diffraction (XRD) and Raman. The carbon soot was processed in a SPEX mill 8000D (SPEX SamplePrep, Metuchen, NJ, USA) for different times (from 1 to 5 h). The milled soot was used as reinforcements for the Si particles to form a composite. The Si-C blend was milled for different times from 1 to 3 h. This new blend is milled until a homogeneous mix is completed and a composite is formed.

### Material characterization

XRD was carried on a D5000 SIEMENS diffractometer, with a Cu tube and a characteristic *K*_*α*_ = 0.15406 nm operated at 40 kV and 30 A. The scanning electron microscopy (SEM) observations were carried out on two field emission SEMs. One is a FEI XL-30FEG and the other is a FE-SEM, Zeiss Supra 40 (Zeiss, Oberkochen, Germany), connected to an energy dispersive X-ray spectroscopy (EDS-Oxford Inca Energy 450, Oxford Instruments, Abingdon, UK). The high-resolution transmission electron microscope (HRTEM) observations were carried in a Jeol 2000FX apparatus, operated at 200 kV. The images were analyzed in DigitalMicrograph 3.7.1 software.

The X-ray photoelectron spectroscopy (XPS) was conducted on a Physical Electronics XPS Instrument Model 5700, operated via monochromatic Al-K_α_ X-ray source (1486.6 eV) at 350 W. The data analysis was conducted on Multipak™ software (Physical Electronics, Inc, Chanhassen, MN, USA), and the Shirley background subtraction routine had been applied throughout. The raw powder was analyzed using a × 1,000 objective lens to focus the laser beam on sample surface, and the size of the focused laser spot on the sample has a diameter of a few micrometers. The Raman system is a confocal micro-Raman XploRA™, Horiba JY (New Jersey, NJ, USA) using a Raman excitation green laser of 532 nm at × 1,000 magnification.

### Battery cell fabrication

#### Procedure

A binder solution is made by mixing 2.0 gm of polyvinylidene fluoride (PVDF) polymer in 10.0 ml of dimethyl formamide (DMF) solvent. The PVDF attaches to C and Si particles via weak van-der-Waals forces. The mixing of polymer is complete in 2 h. A second solution of carbon-based material is made by dissolving 1.0 gm of CNS or CNS-Si in 20 ml of DMF solution. The mixture is stirred for 20 h and then sonicated for 4 h. The above two solutions are mixed and further stirred for several hours at room temperature and finally sonicated for 1 to 2 hs before use as coating on nickel strips. The strips of nickel foam are cut in exact dimensions (usually 2 × 7 cm) and are weighed individually and labeled. These foam strips are washed thoroughly by soaking in acetone and rinsed with fresh acetone and oven-dried at 150°C. The weight of each strip is recorded before they are being coated.

#### Anode fabrication

For anode fabrication, first, nickel strips are dipped in the prepared coating mixture above and dried in air. Air-dried strips are mechanically pressed and further dried in air and finally in a hot oven (100°C). The weight of each dried strip is recorded. These strips are coated again, drying steps were repeated, and weights are recorded. Strips are pressed one more time and coated again and completely dried in air and hot oven. Heat treatment of PVDF-based CNS-Si anodes under argon atmosphere has been found to significantly improve the binder's adhesion to both CNS-Si particle-coated nickel strips and to the copper foil current collector, resulting in improved stability of the battery during cycling [30]. The final weight of each strip is recorded. These strips are used in battery assembly.

#### Cathode fabrication

Electrodes were prepared with LiCoO_2_ powders, PVDF (Aldrich, Wyoming, IL, USA) as binder and carbon black (MTI) at the 85:5:10% *w*/*w* ratio, using (DMF) (Aldrich) as solvent. The mixture was sonicated for 8 h for the formation of a homogeneous solution. The mixture was painted on Aluminum films (100 μm) and, in order to evaporate the solvent, the electrodes were dried at 120 C for 24 h in vacuum.

#### Battery pouch fabrication

Pouch-type cells were assembled in Glovebox under argon atmosphere. As separator, polyethylene with thickness 16 ~ 25 μm, surface density 10 ~ 14 g/m^2^, porosity 36 ~ 44%, pore size 0.01 ~ 0.1 μm, mainly 0.03 μm, penetration strength 0.5 ~ 0.65 kg/mm, tensile strength <600 N/m, and shut-off temperature 131 ~ 133°C was used. The electrodes were immersed in nonaqueous electrolyte (1 M LiPF_6_ in ethyl carbonate/dimethyl carbonate 1:1) for 12 h, after which the pouch cell was hermetically sealed in laminated aluminum case and tested.

### Electrochemical characterization

The fabricated anodes along with a commercial one were integrated and tested with matching commercial cathode materials; both anode and cathode are available from MTI Corporation (Richmond, CA, USA). For quick laboratory assessment, all electrochemical cells were fabricated under available environment conditions and they were tested at room temperature only. The tests were performed with eight channel battery analyzer (MTI) under constant current-constant voltage charging mode and constant current discharging mode. All cells were tested at room temperature. The loading density of electrodes was 15 to 20 mg/cm^2^. All cell tests had 1 min open-circuit rest at the end of each charge and discharge.

## Results and discussion

**Figure 1:**
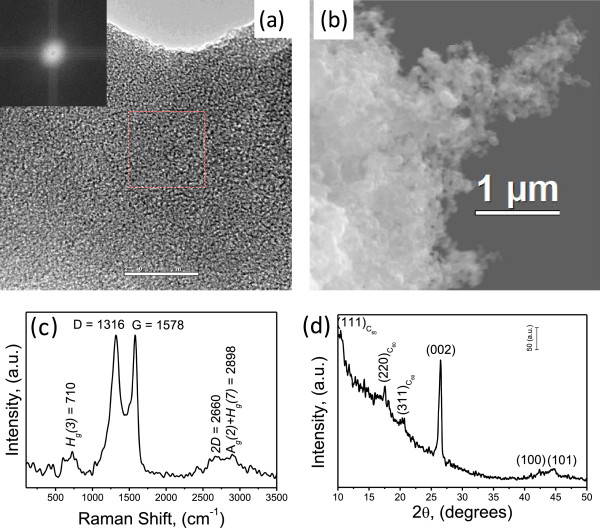
**The carbon soot characterization.** Characterization of the fullerene soot in raw conditions by the following methods: **(a)** Raman, **(b)** XRD, **(c)** HRTEM, and **(d)** SEM.

The carbon soot characterization is presented in Figure 1a,b where it is possible to observe that the carbon soot has a fluffy appearance and has an amorphous nature. This is typical of an evaporated material and is confirmed by SEM and HRTEM. The XRD results have two main characteristics, the presence of the C_60_ and the (002) graphite reflection. The presence of C_60_ are leftovers in this byproduct; In highly efficient methods are obtained 14.5 g or more of soot per each gram of fullerene that results in significant price reduction. The pricing of this material is as affordable as carbon black. Therefore, if there is detectable amounts of C_60_, they are the leftovers and never exceed more 1 wt% of C_60_ making its identification with both XRD and Raman hard (Figure 1c,d). The square in the dotted lines in (d) identifies the location where the FFT-diffraction patter (inset) was made. The soot is the waste on this synthesis and it is our raw material. Additionally, this raw material is ideal for thermomechanical processing when it can be transformed into effective reinforcements such as graphene or graphitic carbon. An alternative source that we are currently investigating includes chimney soot.

**Figure 2:**
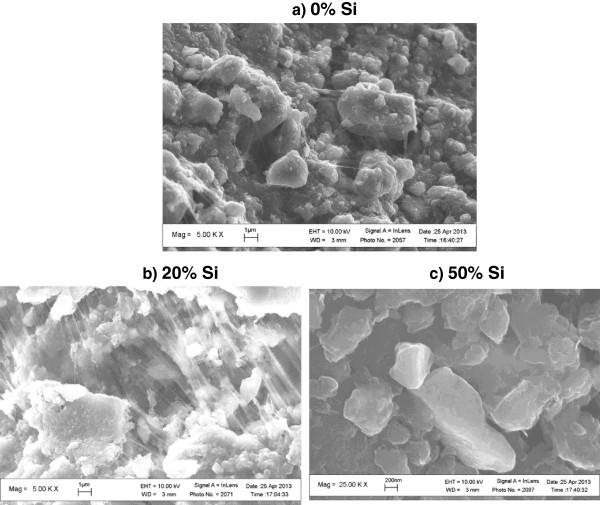
**SEM of the investigated anodes embedded in the polymer or binder (PVDF). (a)** pure CNS and **(b,c)** composites containing (b) 20 wt% Si and (c) 50 wt% Si.

In XRD, the (002) reflection indicates the presence of the benzoic groups that are not forming mid- to short-range ordered structures and they are high density of dangling bonds that contributes to the D band at approximately 1,330/cm. The above description matches with the presence of graphitic structures having a high density of defects. An important characteristic of our CNS is its potential to transform *in situ* into effective reinforcements, namely, graphene and graphitic carbon, during mechanical milling. In other words, our carbon soot has the ability to induce phase transformations during processing resulting in the synthesis of effective reinforcements that have positive effects on mechanical characteristics that are key for batteries.The SEM micrographs presented in Figure 2 show the composite structures of silicon embedded in carbon nanostructures. In this case, the carbon is acting as a coating over the silicon nanoparticles. This combination is expected because of the high elastic properties of the graphene and graphitic structures that are part of the carbon nanostructures. The rest of the composite is the polymeric binder that is discernible by its fiber appearance. The binder is used to hold the silicon-carbon nanostructures together. The concentration of silicon is evident and the composite with 50 wt% Si clearly shows the presence of a large amount of highly crystalline particles. The silicon is obtained from wafers that are milled to sub-micrometric and nanometric sizes to improve their surface area and hence efficiency to collect lithium.

**Figure 3:**
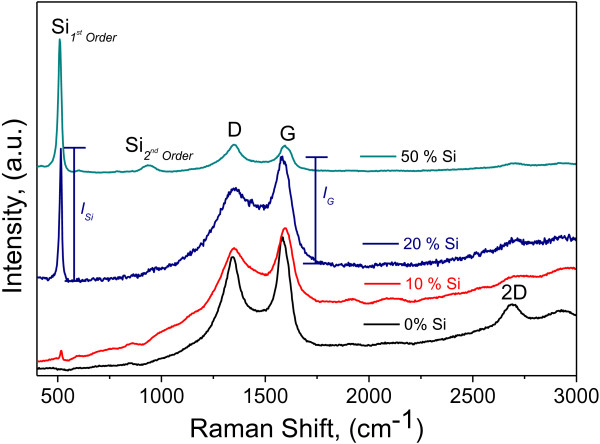
**Raman analysis of CNS-Si at different Si contents.** The relative intensities for *I*_*G*_*/I*_*Si*_ are as follows: 0, 0.15, 1.25, and 5.6 for 0, 5, 10, and 50 wt% Si, respectively.

**Figure 4:**
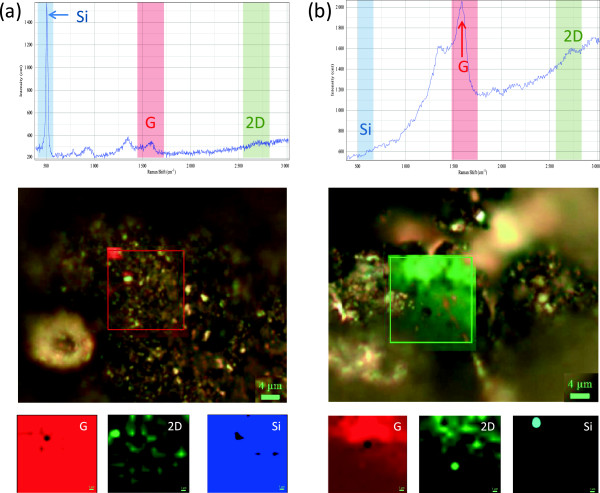
**Raman mapping analysis. (a)** 50 wt% Si and **(b)** 0 wt% Si.

The milled soot shows the 2D band in Raman at approximately 2,700/cm. This feature is typical of graphene or graphitic carbon that is the single most important constituent in our CNS due to its positive improvements in mechanical characteristics (Figure 2). Our interest in those structures is due to their outstanding mechanical properties, in particular, their elastic behavior [31-33]. The particles are formed in times of 10 h or less in a high-energy mill (SPEX).The Raman characterization presented in Figure 3 shows the presence of both constituents in the composite. Silicon can be identified in the 1 wt% Si sample with a relatively small reflection at approximately 521 nm. This reflection intensity increases with Si content; however, this is clear if we considered that the Raman results presented in a normalized scale. Further, the intensity of Si increases proportionally to the Si content that is more evident when the results are analyzed in normalized intensity. We use a × 1,000 magnification in Raman to be able to analyze the material in a discrete fashion with the potential to discern Si and the thin layer of carbon along the Si particles.The results presented in Figure 4 show Raman mapping of the carbon nanostructures and silicon composites. In Figure 4a, the presence of both constituents Si and carbon nanostructures is observed. Due to the higher crystallinity of Si, the Raman spectrum is mainly dominated by the first order band of Si at approximately 521 nm. Nonetheless, the presence of carbon is also discernible in the spectrum. In Figure 4b, pure carbon is observed as no silicon is expected. In both cases, the spectrum shows the D, G, and 2D bands for carbon. The D band is also known as defect band that in this case is by the large amount of defects or dangling bonds implying that our carbon is nanostructured; on the other hand, the 2D band is of major importance in this work because this band is the evidence for the presence of graphene and/or graphitic carbon. The presence of this type of carbon nanostructures is responsible for the outstanding elastic behavior of the composite. The mapping demonstrates that our composites are homogeneous and is observed in Figure 4 by the good dispersion of the constituents on the maps. Therefore, we can conclude two things: (i) the carbon nanostructures contain graphene and graphitic structures and (ii) the mechanical milling is an ideal method to manufacture Si-carbon nanostructure composites for rechargeable batteries.

**Figure 5:**
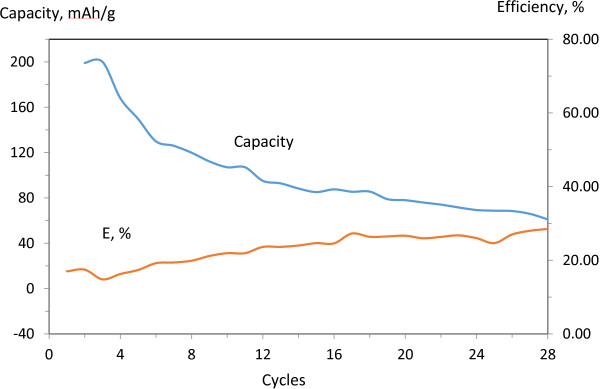
Capacity/efficiency of CNS -0% Si anode-based full cell lithium ion battery.

**Figure 6:**
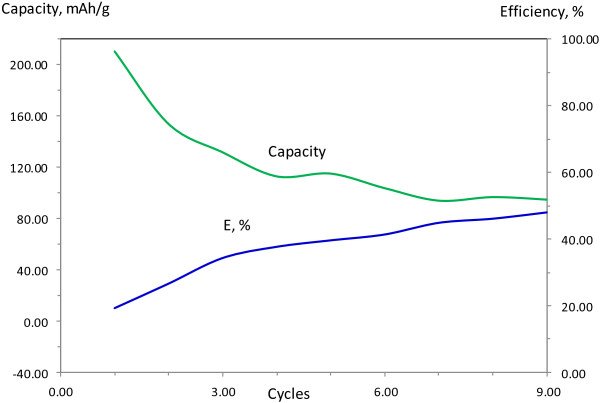
Capacity/efficiency of commercial-activated carbon anode-based full cell lithium ion battery.

**Figure 7:**
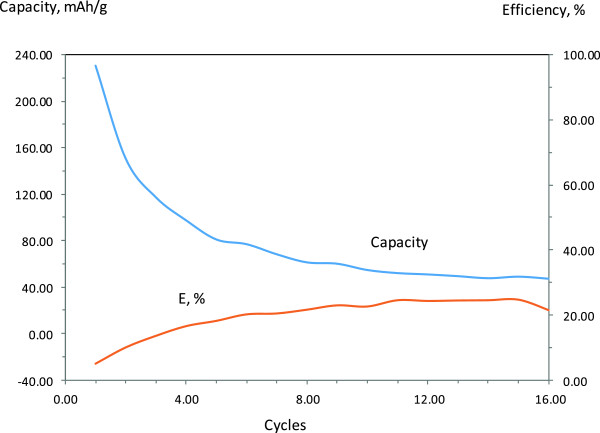
Capacity/efficiency of CNS -20% Si anode-based full cell lithium ion battery.

The electrochemical characterization showing capacity and efficiency along with materials cyclability of the three made battery pouches are presented in Figures 5, 6 and 7. A typical AC anode has a capacity of 372 mAh/g. The cathode which is made of LiCoO_2_ powders has a capacity of 140 mAh/g. This cathode drives the capacity of the cell at 100 mAh/g. The fabricated pouch-type cells are also a cathode-limited cell and shows a capacity about 100 mAh/g. The anode made of CNS material only (Figure 5) shows a reversible capacity of 112 mAh/g after the ninth cycle with a coulombic efficiency (CE) of 21% and stabilize after 28 cycles with a reversible capacity of 61 mAh/g with a CE of 30%. Efficiency is calculated as how successfully the capacitance comes close to the value if there was no capacity loss (100% corresponds to no capacity loss). This battery cell which is made of CNS anode shows more or less similar performance to the commercial one which is made of a copper foil coated with activated carbon. The later stabilizes after nine cycles and shows a reversible capacity of 85 mAh/g with a CE of 48% (Figure 6). Blending Si with CNS was expected to increases the overall capacity of the cell as a result of increasing the capacity of the anode material. Anode material made of blended CNS with 20 wt.% silicon stabilizes after 16 cycles and shows less reversible capacity and efficiency after compared to the previous battery cells (Figure 7). The characteristic of a cell containing 50 wt% (not presented) of silicon shows very poor capacity and efficiency. Lower performance of carbon-silicon-based cells is most likely attributed to the larger size of silicon particles as well as the low electrical conductivity of the hybrid carbon-silicon material as a result of oxidation of the silicon particles during the thermo-milling process.

## Conclusions

The carbon soot has an amorphous nature and milling transforms it into graphene and graphitic carbon. The carbon nanostructures are capable of coating the Si particles promoting a strengthening mechanism that improves the life cycle on the battery. The investigated processing methods and materials are cost effective and demonstrate to be able to produce composites with high homogeneity.

Initial electrochemical analysis results on specific capacity, efficiency, and cyclability of CNS in comparison to currently available activated carbon counterpart are promising to advance cost-effective commercial lithium ion battery technology. The electrochemical performance observed for CNS material is very interesting given the fact that CNS’s production cost is away cheaper than activated carbon. The cost of activated carbon is about $15/kg whereas the cost to manufacture CNS soot as by-product from large-scale milling of abundant graphite is about $1/kg. We believe this technology will boost the performance and stability of the lithium ion batteries while driving the price of actual anode materials down from $20 to $40/kg to about $5/kg. In particular, for stationary energy storage applications, cost along safety is the most important factor to consider. In order for the hybrid CNS-silicon material to show great promise for use in fabricating electrodes for a new breed of low-cost and high-performance lithium ion batteries, the size of silicon particles needs to be refined at the nanometer scale along with a process development to effectively remove the native silicon oxide. To that end, characterization of a half-cell configuration of proposed anodes is being carried out and results will be compared with AC-based anode in terms of specific capacity, efficiency, and degradation using cyclic voltammetry analysis.

## Competing interests

The authors declare that they have no competing interests.

## Authors' contributions

NB conceived the idea and planned the experiments related to the battery anode fabrication. ARE carried out the preparation of the coating material for the anodes. FCHR carried out the structural characterization of the materials and analyzed the data. AOO carried out the synthesis of the materials. KM carried out the battery assembly. MH carried out the electrochemical characterization of the battery cells. NB, FCHR, and KM contributed to the preparation of the manuscript. All authors read and approved the final manuscript.
